# Assessment of hybrid nanocomposite AFOs for pediatric cerebral palsy: mechanical, spectroscopic, and finite element analysis

**DOI:** 10.1038/s41598-025-13344-1

**Published:** 2025-08-07

**Authors:** Noorhan Abdelgawad, Marwa M. A. Hadhoud, Mohamed Tarek El-Wakad, Reda Abdelbaset

**Affiliations:** 1 Department of Biomedical Engineering, Helwan University, Cairo, Egypt; 2 Faculty of Engineering and Technology, Future University in Egypt, Cairo, Egypt; 3 Department of Mechatronics, Canadian International College, Cairo, Egypt

**Keywords:** Ankle foot orthoses, Cerebral palsy (CP), MWCNTs, PLA, Orthocryl composite material, Mechanical testing, Finite element analysis, Material flexibility, Pediatric orthoses, Biomedical engineering, Health occupations, Materials science

## Abstract

Cerebral Palsy (CP) is a neurological disorder that affects motor function and causes gait abnormalities in children. Ankle–Foot Orthoses (AFOs) are external aiding devices that provide stability and improve mobility for pediatrics. However, conventional AFO materials often fail to achieve an optimal balance of strength, flexibility, and energy absorption for dynamic movements. This study introduces a novel composite material for pediatric ankle–foot orthoses (AFOs), based on Orthocryl and reinforced with multi-walled carbon nanotubes (MWCNTs) and polylactic acid (PLA). The proposed formulation is engineered to overcome the limitations of conventional materials by providing enhanced mechanical performance and improved functional suitability for clinical applications. Four composite concentrations were fabricated: pure Orthocryl, 0.5% MWCNTs, 0.5% MWCNTs/1.0% PLA, and 0.5% MWCNTs/1.5% PLA. Mechanical and morphological characterizations were performed using a universal testing machine for tensile, flexural, and impact testing, Fourier Transform Infrared Spectroscopy (FT-IR) for material composition analysis, and Field Emission Scanning Electron Microscopy (FE-SEM) for surface morphology examination. To simulate practical application, Finite Element Analysis was performed using ANSYS software, recognizing gait loading conditions. The experimental findings demonstrated that incorporating 0.5% MWCNT into Orthocryl significantly enhanced its mechanical properties, with a 12.5% increase in tensile strength (from 52.79 to 59.4 MPa), a 59.3% increase in flexural strength (from 52.08 to 82.93 MPa), and a 22% improvement in impact resistance (from 28.12 to 34.3 kJ/m^2^). These improvements confirm the effectiveness of MWCNT reinforcement. Additionally, FE-SEM and FT-IR analyses confirmed the uniform distribution of CNTs within the matrix and stronger interfacial bonding between the filler and polymer. Simulation results showed that the 0.5% MWCNT/1.5% PLA composite had the highest deformation (10.95 mm) with a safety factor of 1.12, indicating acceptable safety. In contrast, the 0.5% MWCNT composite showed the lowest deformation (4.17 mm), 12.6% less than pure Orthocryl, and the highest safety factor (3.2), reflecting an optimal balance of strength and flexibility for pediatric AFOs in CP patients.

**Supplementary Information:**

The online version contains supplementary material available at 10.1038/s41598-025-13344-1.

## Introduction

Cerebral palsy (CP) is the most prevalent motor disability in children, affecting approximately 17 million people worldwide, with higher prevalence in low- and middle-income countries due to inadequate prenatal and neonatal care^1–3^. Drop Foot (DF), which is frequently caused by CP or other neurological problems, is defined by difficulties elevating the foot, resulting in dragging toes while walking. Physical therapy, electrical stimulation, and AFOs are among the treatment options available to improve function and mobility. AFOs are critical biomechanical devices that support the foot and ankle while improving balance and movement in patients with motor impairments^4–6^.

AFO materials have progressed from traditional plastics to advanced composites such as carbon fiber and carbon nanotube-infused polymers^7,8^. These materials provide increased strength, flexibility, and impact absorption, all of which are necessary for boosting AFO effectiveness. However, typical materials such as polypropylene (PP) are limited in stiffness and durability. The use of more modern materials, such as MWCNTs and PLA, offers enhanced mechanical performance, although difficulties such as increased brittleness and expense persist ^9,10^.

AFOs have been manufactured using a variety of materials, including layers of perlons, fiberglass, and composites such as Abaca fiber, epoxy, and activated carbon particles. Laminated PLA with carbon fiber has shown potential in enhancing the flexibility and comfort of CP patients. Carbon fiber orthoses are especially effective for pediatric CP patients and polio survivors, as they improve gait efficiency and energy return^10,11^. MWCNTs have also been investigated for their high mechanical qualities, which boost tensile strength but may diminish elasticity and impact force absorption if employed in excess^12,13^.

PLA a biodegradable polymer, provides excellent flexibility and impact absorption, but its limited strength makes it less suitable for high-stress environments^14,15^. When combined with materials like MWCNTs, PLA can enhance the overall performance of AFOs, improving flexibility and comfort without compromising the material’s structural integrity^16–19^. This dual-material approach promises to optimize both mechanical properties and patient comfort.

FEA offers a powerful, non-invasive method for simulating the mechanical behavior of AFO materials under physiological loading conditions. By using FEA^20,21^, researchers can model stress distribution, deformation, and energy transfer, which assists in evaluating the performance of innovative materials and designs before physical production. This approach not only reduces costs associated with prototyping but also supports evidence-based material selection tailored to the specific biomechanical needs of CP patients.

AFO manufacturing methods, such as casting and additive manufacturing (AM), each have their respective advantages and drawbacks. Casting offers enhanced material flexibility, durability, and mechanical strength, making it suitable for high-stress applications and large-scale production. Moreover, casting provides greater control over thickness, rigidity, and layer compression, enabling the production of lightweight, cost-effective, and high-performance AFOs^22–25^.

Existing AFO materials, such as polypropylene and carbon fiber, often suffer from limitations in mechanical performance, comfort, and environmental sustainability. While advanced materials such as MWCNTs and PLA have shown promise in improving AFO functionality, there remains a gap in understanding their potential when combined in composite materials and processed through vacuum casting lamination. Additionally, the integration of MWCNTs into AFO manufacturing requires further investigation to optimize the material’s mechanical and functional properties for improved patient outcomes.

This study aims to develop and characterize MWCNTs/PLA/Orthocryl composite materials intended for use in AFOs using vacuum casting lamination. The primary focus is to enhance the mechanical and functional properties of MWCNT-based composites, creating lightweight, durable, and environmentally sustainable AFOs that improve patient comfort and mobility. This will be achieved by fabricating composite materials with varying MWCNTs and PLA concentrations (0.5% MWCNTs, 0.5% MWCNTs/1% PLA, and 0.5% MWCNTs/1.5% PLA), based on concentrations used in prior research. Mechanical properties will be assessed through tensile, flexural, and impact testing, while material composition will be analyzed using FT-IR, and surface morphology will be examined using FE-SEM. The experimentally measured material characteristics have been imported into the FEA of the AFO model to simulate stress distribution and deformation, allowing for a thorough assessment of their suitability for orthotic applications.

## Materials and methodology

This study involves the fabrication and characterization of composite materials made from PLA, MWCNTs, and Orthocryl (polymethyl acrylate resin) for use in AFOs. The materials were systematically prepared and combined through a lamination process to produce specimens with varying concentrations. The following section outlines the detailed steps of material preparation, lamination, specimen casting, and characterization techniques, including mechanical testing, FE-SEM, and FT-IR analysis.

### Materials preparation

**Fig. 1:**
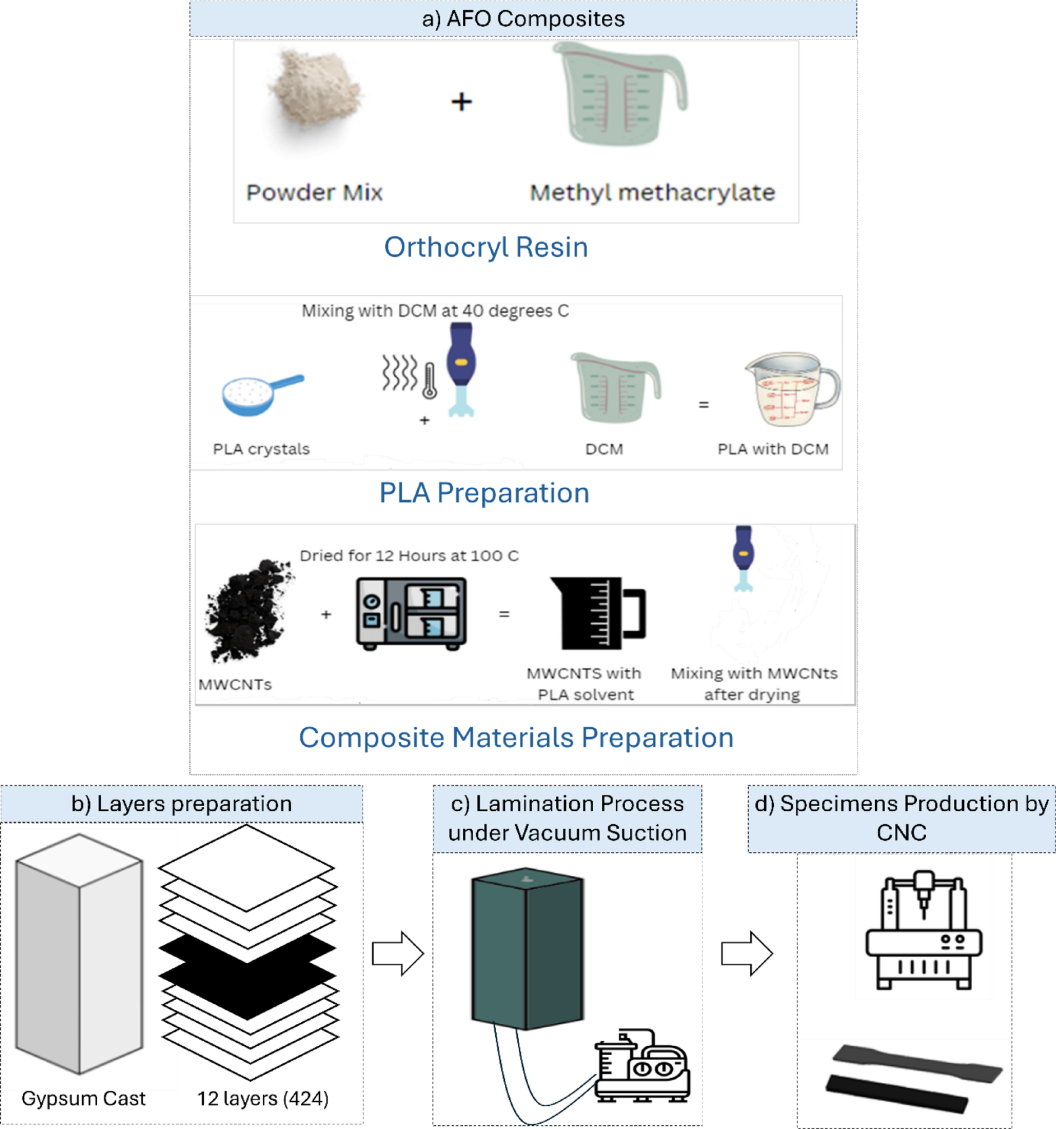
The process for materials preparation and lamination Process, (**a**) AFO composite’s Preparation, (**b**) Layers preparation (4perlons, 2Carbon fibers, and 4 perlons, (**c**) Lamination process under vacuum suction, and d) Specimens production by CNC.

The preparation of the composite materials was carried out through a systematic layering and lamination process to ensure structural consistency and mechanical reliability. Figure 1 outlines the key steps, including materials mixing sequence preparation, layer arrangement, vacuum-assisted lamination, and CNC-based specimen fabrication.

#### Orthocryl lamination resin

As shown in Fig. 1, the used resin in the lamination process contains a solution mix of (Methyl methacrylate (40–70%), Tolyiminodipropan-2-ol (0.1–1%)), and a powder mix of (Dibenzoyl peroxide (30–60%), Dicyclohexyl phthalate (30–60%). The powder mixture is combined with the solution at a 1:3 ratio (powder to solution) by volume. The interaction process increases with higher powder-to-solution ratios. The Orthocryl used in the experiment specializes in orthoses and prostheses with the mark of 17H119 Orthocryl lamination Resin.

#### PLA preparation

PLA (PLA 6202D, Natureworks LLC) is dissolved in DCM (Dichloromethane) under 40 °C with mixer 1400 rpm^26,27^. DCM was selected as the solvent due to its low boiling point, which does not exceed 40 °C, thereby preventing thermal degradation of PLA during processing.

**Fig. 2:**
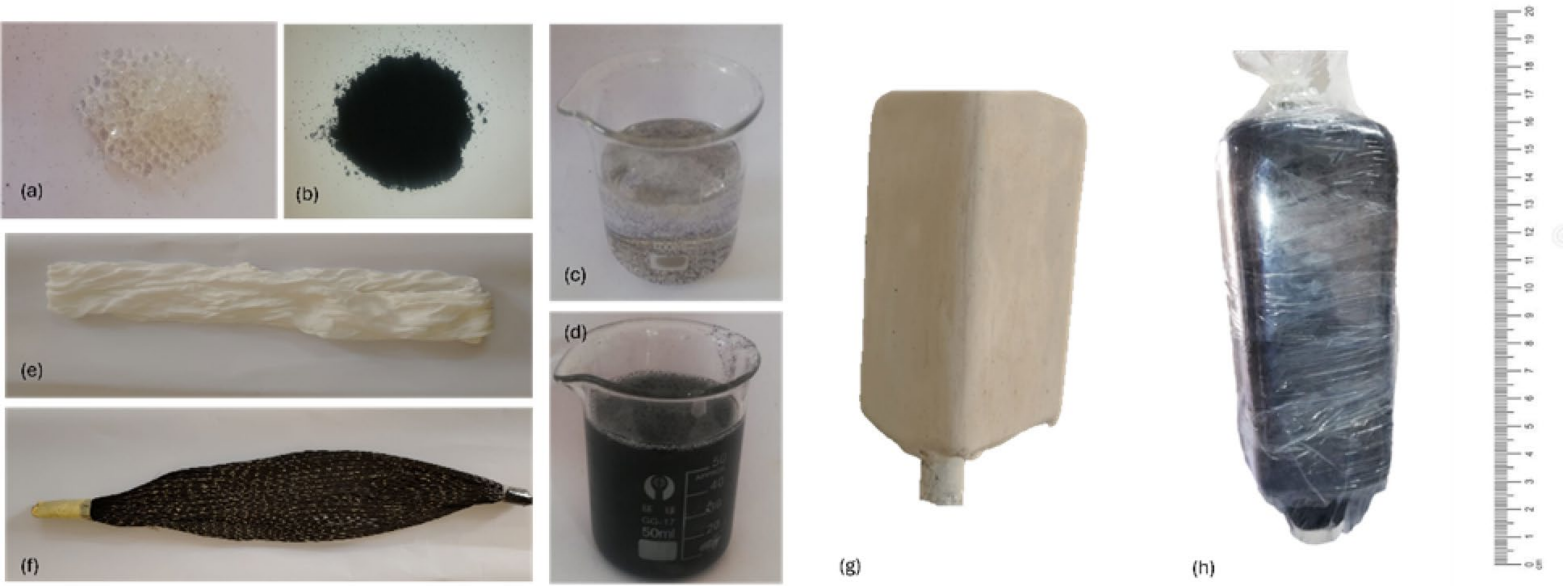
Process of the material preparation and casting; (**a**) PLA crystals, (**b**) MWCNTs, (**c**) solvent of the PLA in DCM powder, (**d**) The solvent of MWCNTs, (**e**) White perlons, (**f**) Carbon Fiber sheets , (**g**) The rectification of the gypsum cast, (**h**) The cast after laminated with cast after laminated with resin and MWCNTs.

Fig. 2a,c) show the crystals of PLA before and after resolving.

#### Composite material preparation

In Fig. 2b, MWCNTs with a length of $100 \mu m$ and diameters ranging from 2 to 40 nm were obtained from Nano Gate Inc. To ensure the removal of moisture, the MWCNTs were dried in an oven at 100 °C for 12 h. The dried nanotubes were then incorporated into PLA using a solvent-assisted mixing process at a speed of 1400 rpm. The resulting nanocomposite mixture is depicted in Fig. 2d. After mixing, the composite was subjected to further solvent removal through oven drying for 3 h, following the procedure described in the reference^28^.

White Perlons, specifically manufactured for orthotic and prosthetic applications, were used in the lamination process, as shown in Fig. 2e, f. The layer sequence consisted of four layers of white Perlons, followed by two layers of carbon fiber sheets, and then another four layers of Perlons, consistent with the standard lay-up techniques employed in orthotic fabrication^29^. After vacuum lamination, the total thickness of the composite reached approximately 4 mm.

Table 1 presents the compositions of the developed samples, detailing the proportions of Perlons, carbon fiber sheets, PLA, and MWCNTs. Four sample groups were prepared. The first group served as a control sample, composed of Orthocryl resin reinforced with Perlons and carbon fiber sheets without the addition of CNTs or PLA. The second group contained 0.5% of MWCNTs, while the third group combined 0.5% MWCNTs with 1% PLA. The fourth group was prepared by adding 0.5% MWCNTs with 1.5% PLA.

**Table 1:** Concentration of MWCNTS, PLA, and Orthocryl lamination resin with perlons and carbon fiber sheets.

Sample with percentage				
Sample	Pure	CNT 0.5%	CNT 0.5%/ PLA 1%	CNT 0.5%/ PLA 1.5%
Orthocryl 3% & powder mix	100%	99.50%	98.5%	98%
MWCNTs	0	0.5%	0.5%	0.5%
PLA	0	0	1%	1.5%

### Lamination method

The manufacturing of the composite samples in this study followed standard lamination techniques typically used in the production of orthoses and prostheses^30,31^. Initially, a negative mold was created using gypsum, after wrapping the prototype with plastic film. Subsequently, a positive mold was cast from the negative impression. Figure 2g illustrates the prepared gypsum mold after surface smoothing, which is an essential step to minimize surface defects and artifacts during lamination.

Once the mold was prepared, the first layer of polyvinyl alcohol (PVA) was applied to its surface. Following this, the reinforcement layers consisting of Perlons and carbon fiber sheets were placed sequentially on the mold. A final PVA layer was then wrapped around the entire assembly to enclose the laminate structure.

Figure 2h shows the final setup during the resin impregnation stage. At this step, MWCNTs, PLA, and powder catalyst were mixed thoroughly and then poured into the PVA-covered layers under vacuum conditions. The lamination process was conducted using a vacuum system operating at a capacity of 20–300 m^3^/h, with a rotational speed of 1440 rpm and an ultimate vacuum pressure below 0.5 bar. After curing, the samples were cut precisely using a laser cutting machine according to the specific ASTM standards required for each mechanical characterization test.

### Material characterization

The material characterization process involved a series of mechanical tests, including tensile testing, three-point bending, and impact testing, to evaluate the strength and energy absorption capacity of the developed composites. Furthermore, morphological analysis was performed using FE-SEM to investigate the fracture surfaces and assess the dispersion of the nanoparticles within the matrix. Complementary to this, chemical characterization was carried out using FTIR to examine the chemical bonding and molecular structure of the composite materials.

#### Tensile test

**Fig. 3:**
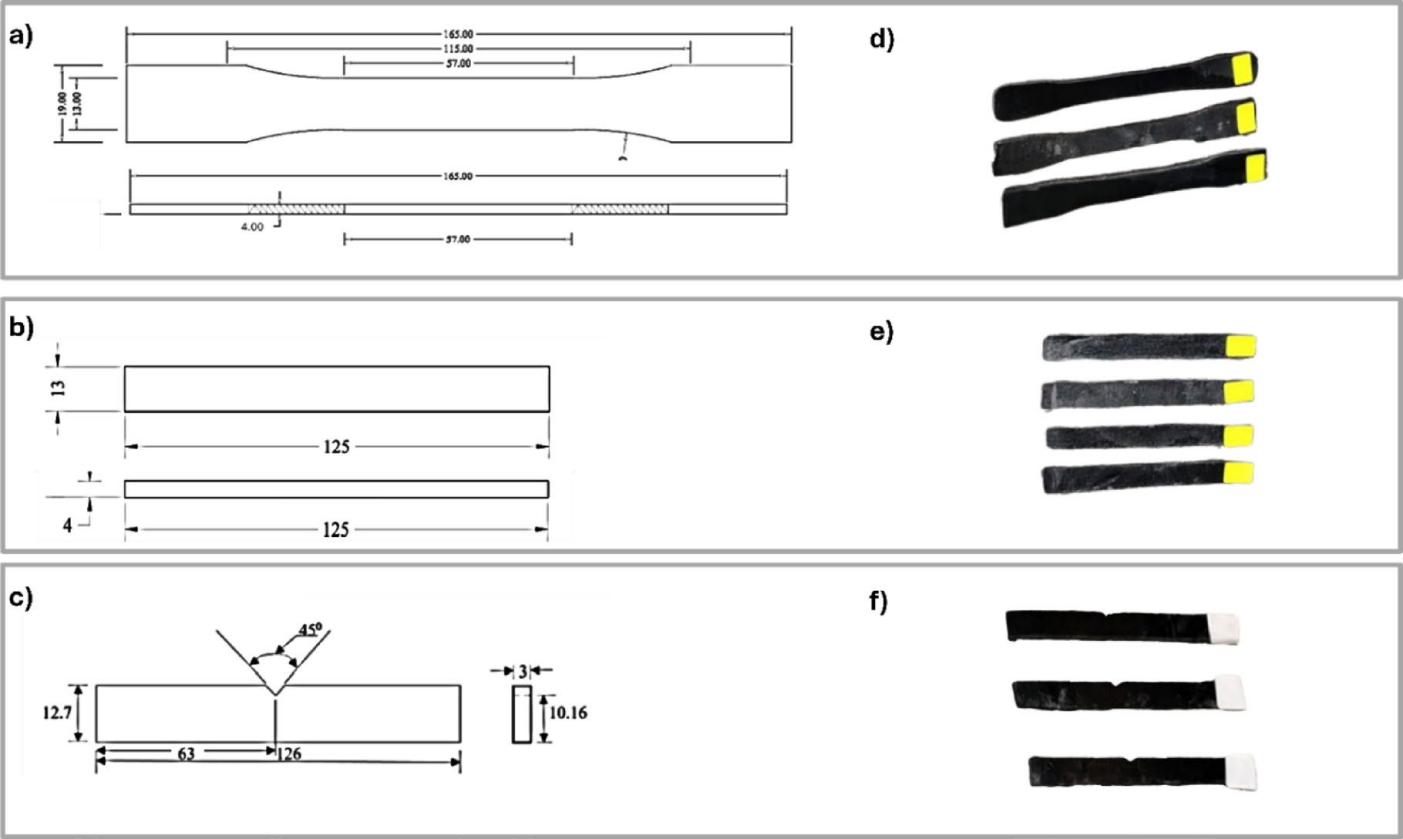
Sample Dimension, (**a**) Tensile test specimen referred to ASTM D638 Type I, (**b**) Bending Test sample referred to D790, (**c**) Impact test specimen referred to ASTM D6110, (**d**) 3D Tensile specimen, (**e**) 3D Bending Specimen, (**f**) Impact test specimen referred to ASTM D6110, (**f**) 3D Impact specimen*.*

**Fig. 4:**
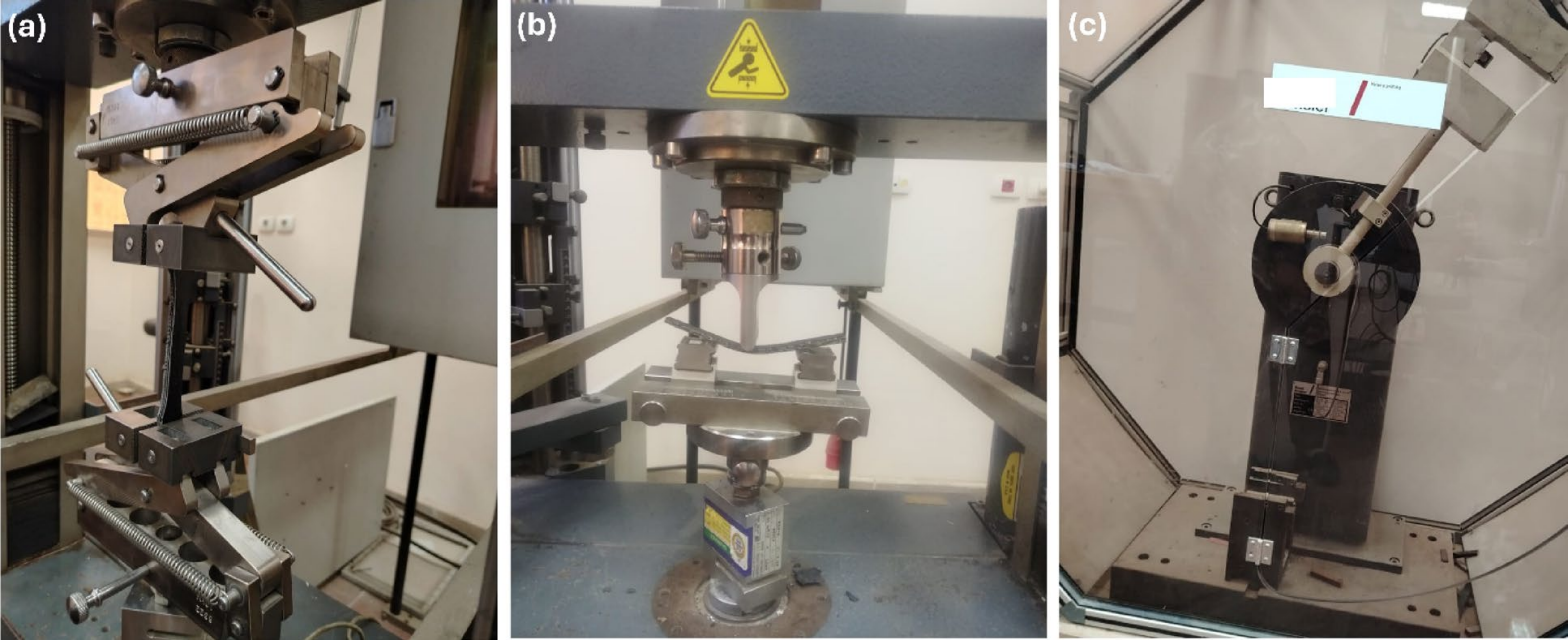
(**a**) Tensile Test, (**b**) Bending Test, and (**c**) Impact Test,

The tensile test is a crucial evaluation used to assess the material’s functionality and durability in orthotic applications. It determines whether the material can withstand the mechanical loads imposed by patients during activities such as walking and running. The tensile test is carried out by a Zwick Roell machine with a maximum load capacity of 10 kN. All tests were conducted under displacement control, with a constant crosshead speed of 2 mm/min following ASTM D638. ASTM D638 was selected as it aligns with the properties and fabrication method of composite material, which involves Orthocryl with dispersed MWCNTs powder prepared through a lamination casting technique. This standard effectively evaluates the mechanical behavior of such polymer-based composites. Tensile strength, Elastic Modulus, and standard deviation are measured. In addition, the average value of three replicates was used to measure the fracture and yield strengths. Displacement and strain measurements were obtained from the Zwick machine’s crosshead movement. Figure 3a illustrates sample dimensions as it was manufactured with ASTM D638 with grips of 65 mm Type I and Fig. 3d shows the tensile specimen. All samples’ dimensions are manufactured by a CNC laser cutting machine. Figure 4a illustrates the specimen tensile machine.

#### Flexural test

The flexural strength of the specimens was determined according to ASTM790 standard using a Zwick universal testing machine equipped with a three-point bending jig. The fixture comprised two cylindrical lower supports and a central upper loading nose, both conforming to the standard’s geometric requirements. Specimens were positioned horizontally on the supports, and the load was applied at the midpoint to induce bending stress. Figure 3b indicates the dimension of ASTM790 where bending samples are cut. The bending specimens are cut by a laser CNC machine with dimensions of 13 mm width, 125 mm length, and a thickness of 4 mm. Figure 3e shows the bending specimens. Figure 4b shows the specimens tested with a Zwick Roell machine with a maximum load capacity of 10 kN. All tests were conducted under displacement control, with a constant crosshead speed of 5 mm/min. The bending strength is calculated from:

1 $${\text{Bending}}\;{\text{Strength}} = \frac{{3 \times {\text{F}}_{\max } \times {\text{L}}}}{{{\text{b}} \times {\text{d}}^{2} }}$$

where ${F}_{max}$ is the maximum applied force, L is the length of the specimens in mm, b is the width, and d is the thickness of the specimen in^32,33^

#### Charpy impact test

Impact tests assess the material or device’s ability to absorb shocks without delivering excessive force to the patients, thereby protecting joints and tissues. The computerized test was utilized to determine the impact performance of the laminated samples. Figure 3c describe the dimension of the Charpy sample with 126 mm length, 12.7 mm width, and a thickness of 3 mm, while Fig. 3f illustrates the impact specimens. The support span was 70 mm, and the capacity of the pendulum was 50 Joules. The v-notch is in the center of the specimen and has an opening angle of 45°. The specimens were cut with a Laser cutting machine. The impact strength energy is calculated^34^. The test specimen was held horizontally using grippers and broken with a single pendulum swing. The specimen’s toughness and ductility were assessed by measuring the energy absorbed before fracture, expressed in J/m. Stronger materials absorb more energy than weaker materials. The test is carried out by the Roell Amsler machine in Fig. 4c according to ASTM D6110^35^.

#### Fourier transform infrared (FT-IR)

FT-IR was used to identify functional groups and assess the chemical interactions between the MWCNTs, PLA, and the Orthocryl matrix. This technique was employed to evaluate the presence of interfacial bonding and confirm the structural integrity of the composite materials, as it is critical for load transfer. FT-IR is applied using a Bruker ALPHA II device with an IR Affinity-1 at room temperature, ranges from 500 to 4000 cm^−1^ mid-IR source and a KBr beam splitter is used in the test. (BOMEM FT-IR spectrometer; MB 147, Canada on KBr disk) system. The size of the prepared samples was observed through dynamic light scattering (DLS, Malvern Zeta sizer nano series, UK). The results were obtained with a universal attenuated total reflectance.

#### Field emission scanning electron microscopy (FE-SEM)

The morphology, structure, and interfacial adhesion between the matrix filler and polymer were examined using a field-emission scanning electron microscope (Quanta FEG) equipped with energy-dispersive X-ray spectroscopy (EDX). An accelerating voltage of 30 kV was applied, and magnifications ranging from 250× to 20,000× were utilized to observe the nanocomposite particle morphologies. An X-ray diffraction model of the nanoparticles was achieved utilizing a “PAN Analytical X’pert High Score Plus” diffractometer, through a Cu Kα emission (30 mA and 40 kV) over the (2θ) series of 10°–90° with a scanning speed of 2° min^−1^. Samples of width 10 mm, length 10 mm, and 4 mm thickness were tested. The normal and cracked surfaces were coated with a small layer of gold before image analysis and observation^17^.

### Finite element assessment

Static structural analysis was conducted using the Static Structural module in ANSYS Mechanical (Workbench 2021 R2), which employs the Mechanical APDL solver. Material properties were assigned based on experimental data. Geometry was prepared and imported into Space Claim, followed by meshing and application of boundary conditions. The major outcomes of the simulation analysis of AFOs include stress distribution, strain, displacement, safety factor (SF), deformation patterns, and rotational stiffness, as well as the material’s performance under physiological loading conditions.

These findings are critical for understanding the mechanical behavior of AFOs and ensuring they provide enough support while maintaining patient stability. The stress and strain results aid in identifying possible failure zones and high-pressure regions. The displacement and deformation patterns are critical for determining the orthosis’s flexibility and capacity to accommodate natural foot and ankle movements during movement. Furthermore, the SF was calculated to evaluate the structural integrity of each composite material in the context AFO design. The SF was calculated as the ratio of the experimentally measured tensile yield strength to the maximum Von Mises stress obtained from finite element simulations under physiological gait loading cases.

The deformation results from the simulation were used to calculate the rotational angle of the AFO cuff and the corresponding rotational stiffness. Rotational stiffness quantifies the resistance of the AFO to bending, directly affecting how much the orthosis allows or restricts ankle movement. Higher rotational stiffness contributes to greater postural support and stability for the user, helping maintain balance during standing and walking^36^. The rotational stiffness was calculated using the applied moment divided by the measured angular deflection according to:

2 $${\uptheta } = \tan^{ - 1} \left( {\frac{{\text{x}}}{{\text{r}}}} \right)$$

3 $${\text{Rotaional}}\;{\text{Stiffness}} = \frac{{{\text{Force}} \times {\text{r}}}}{\theta }$$

where θ is the angle deflection, x is the highest deformation in cuff loading simulation, r is the AFO height, and F is the applied force.

This approach accounts for material performance under expected biomechanical stresses and serves as a benchmark for comparing the load-bearing reliability of each formulation^30,31^. The results compared with^37^ to assess the results where the results comparing them with the experimental data to assess the proposed materials to be applied in orthoses with their needed applications^38^.

#### AFO design selection & geometry

**Fig. 5:**
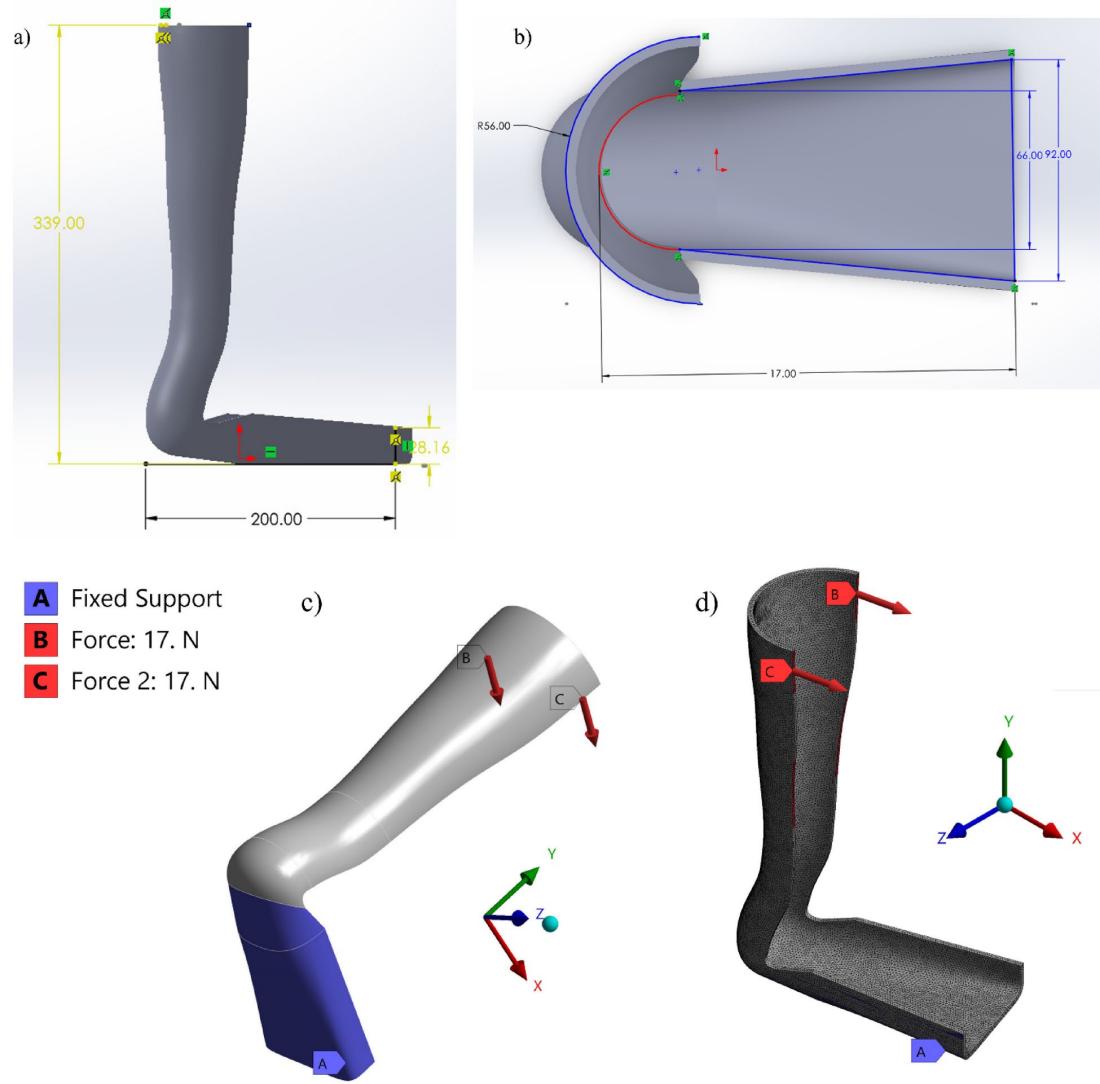
(**a** & **b**) AFO Geometric Dimensions, (**c**) AFO’s Boundary Conditions, and (**d**) Meshing 2.5 mm element Size.

The AFO design shown in Fig. 5a, b was customized for a 9-year-old female patient with CP, featuring key dimensions such as a total height of 339 mm, a footplate length of 200 mm, and a heel cup radius of 56 mm. The initial geometry was obtained from the GrabCAD online repository^39^ and imported into SolidWorks 2023 for dimensional adjustments and measurements. The modified model was then transferred to ANSYS Workbench 2021 R2 to perform static structural analysis and evaluate stress distribution and deformation under representative loading conditions.

#### Material properties input

Material characterization was performed for four different compositions: Pure Orthocryl, CNT 0.5%, CNT 0.5% / PLA 1%, and CNT 0.5% / PLA 1.5%. Key mechanical properties such as Young’s modulus, yield strength, tensile strength, flexural strength, Poisson’s ratio, and fracture energy density were experimentally determined. Poisson’s ratio was calculated using the standard relation between lateral and longitudinal strain. The initial and final dimensions of each specimen were measured using digital calipers before and after the tensile test. Longitudinal strain was obtained from the change in gauge length, while lateral strain was determined from changes in specimen width. These values were used to calculate Poisson’s ratio for each sample to be used in the FEA to approximate the material’s behavior. Table 2 shows these properties, which serve as inputs to the finite element model for assessing the mechanical response of each material in the AFO design.

**Table 2:** Experimental measured mechanical properties of materials for fea

Property	Pure	CNT 0.5%	CNT 0.5%, PLA 1%	CNT 0.5%, PLA 1.5%
Young’s modulus MPa	2369.5	2416.2	1383.3	921.3
Yield strength MPa	50.3	58.9	24.4	20.6
Poisson’s ratio (ν)	0.193	0.186	0.1804	0.1948
Tensile strength MPa	52.79	59.4	23.81	21.95
Flexural strength MPa	52.07	82.92	42.95	38.29
Density g/cm^3^	1.24	1.8	1.81	1.81
Fracture energy density MJ/m^3^	6.86	8.37	10	10.57

#### Mesh refinement

The mesh division approach improves the accuracy of findings from the finite element method^40^. 3D meshing was generated using ANSYS software (Version 21.0, Canonsburg, USA) with an adaptive meshing function and element sizes ranging from 2 to 4 mm. Compared to previous studies that utilized a 4.8 mm element size^41^, an optimal element size of 2.5 mm was selected, as it yielded results with less than a 5% difference. Table 3 presents the mesh refinement process, which was guided by a convergence test.

**Table 3:** Mesh sensitivity study.

Iterations	Element Size (mm)	Nodes	Elements	Max von mises stress (MPa)	Max. strain (mm)	Computational time (min.)
1	2	443,821	280,256	17.98	4.89	45
2	2.5	232,127	139,903	17.45	4.77	30
3	3	152,591	90,460	17.46	4.249	28
4	4	62,572	33,576	16.32	4.22	25

The final mesh was generated following convergence testing, employing coarse span angle center control. The mesh consists of 232,127 nodes and 139,903 elements, utilizing 10-node quadratic tetrahedral (Tet10) elements to accurately capture the complex geometry of the ankle–foot orthosis. A full integration scheme was employed, and hourglass control is not applicable due to the use of second-order elements.

A mesh quality assessment was conducted to ensure numerical reliability and solution accuracy. The element quality ranged from 0.16 to 0.99, indicating generally well-shaped elements across the domain. The Jacobian ratio was within the range of 0.29 to 1.0, Skewness values ranged from 0.2 to 0.9, and the aspect ratio varied between 1.1 and 10.0. Element quality was selected as the primary indicator in this study due to its comprehensive representation of element shape performance in solid FEA. The final model with meshing is illustrated in Fig. 5d.

#### Load & boundary constrains

FEA was performed using ANSYS Workbench 21 to evaluate the structural performance of the proposed composite materials in the AFO design prior to clinical validation. The simulation was conducted under static loading conditions to replicate the behavior of orthosis during standing. As illustrated in Fig. 5c, a total load of 34 N was applied to the model, divided into two symmetrical forces of 17 N each, directed along the X-axis and positioned on the medial superior and lateral superior sides of the ankle. This loading condition simulates the tension exerted by the orthosis straps in an anteroposterior direction during use. The force magnitude of 34 N was identified as the minimum load required to induce significant rotational deformation in the AFO model^41^.

#### Finite element simulation

All finite element simulations were performed on a personal computer equipped with an 11th Gen Intel® Core™ i7-1165G7 CPU @ 2.80 GHz, 8 GB RAM, and a 64-bit Windows 10 operating system. The model consisted of 232,127 nodes and 139,903 Tet10 elements, with a global element size of 2.5 mm. Each simulation run required approximately [30 min] to complete in ANSYS Mechanical, depending on loading and solver settings.

## Results

The effect of adding MWCNTs, PLA, and Orthocryl with casting processes is illustrated in this section. First, the mechanical test results are shown. Tensile, Flexural, and Charpy impact tests are demonstrated. Then, an analytical analysis of sample composition is shown by Fourier transform infrared and field emission scanning electron microscopy. The FE-SEM images the top structure of materials bonding before and after the crack.

### Mechanical test

**Fig. 6:**
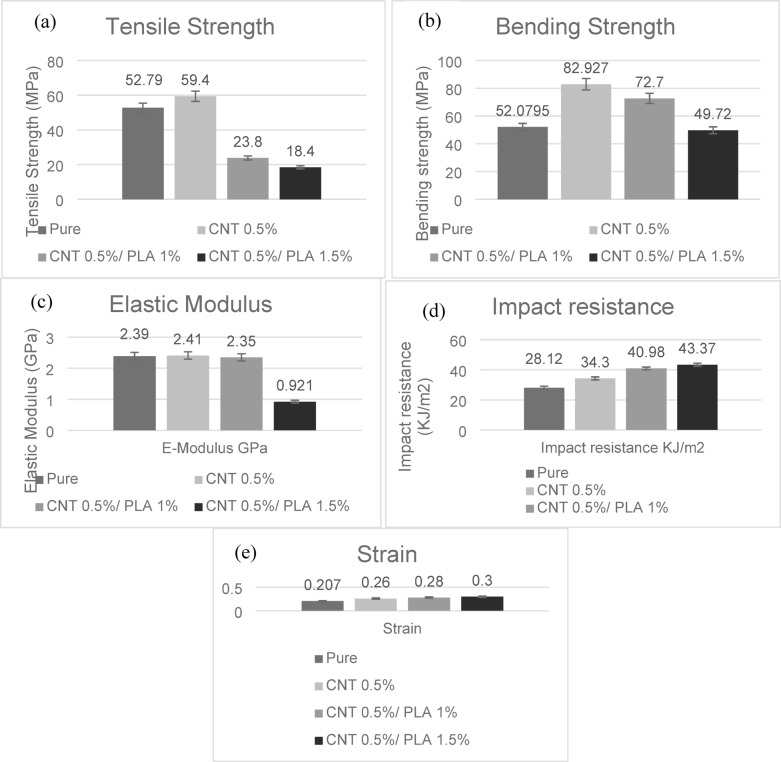
The Mechanical Test Results for four specimens, (**a**) Tensile strength in MPa, (**b**) Flexural strength in MPa, (**c**) Elastic Modulus, (**d**) Impact resistance, (**e**) Strain in Specimens.

The variation in stress strength that corresponds to the tensile strength of the composite laminate is shown in Fig. 6. It was observed that the material with 0.5% CNT has the highest strength among all specimens, whereas the mixed specimens, with 0.5% MWCNTs and 1.5% PLA, have the lowest tensile strength. Figure 6a shows the tensile strength results with the average strength of all specimens being 52.9, 59.4, 23.81, and 21.95 MPa with pure, CNT 0.5%, CNT 0.5%/PLA 1%, and CNT 0.5%/PLA 1.5%, respectively. The addition of PLA at varied concentrations resulted in reduced strength. The tensile strength decreases as the concentration of PLA increases.

The flexural test is applied with three-point bending conditions. The stiffness of the material is shown by the flexural strength obtained from these tests. AFOs need to have the right amount of rigidity to support while also being flexible enough to permit natural mobility. In Fig. 6b, it was observed that the average flexural strength is 52.07, 82.92, 42.95, and 38.29 MPa with pure Orthocryl, CNT 0.5%, CNT 0.5%/PLA 1%, and CNT 0.5%, respectively. Figure 6c illustrates the elastic modulus where it decreases with increasing PLA concentrations to 0.921 GPa, while the pure Orthocryl, CNT 0.5%, CNT 0.5%/PLA 1%, and CNT 0.5/PLA 1.5% have 2.39, 2.41, and 2.35 GPa, respectively. CNT 0.5% has the highest flexural strength, while adding PLA with 1.5% concentration has the lowest flexural strength. The decrease in strength is acceptable, as it promotes greater elastic deformation and flexibility. Figure 6e illustrates the increment of strain when adding PLA with MWCNTs and Orthocryl resin, which allows the material to elongate under stress more than a composite with only Orthocryl resin.

Each composite laminate goes through an impact test to determine its performance. Orthoses, especially AFOs, are frequently subjected to dynamic stresses during running or walking. The impact absorption of energy reached its maximum at CNT0.5/PLA 1.5% with 43.4 $\text{KJ}/{\text{m}}^{2}$. The absorption energy is 28.12, 24.3, 40.9, and 43.4 $\text{KJ}/{\text{m}}^{2}.$ Figure 6d compares composite specimens with and without nanofillers; the impact strength of specimens with nanofillers MWCNTs/PLA has significantly improved, as it got the highest impact energy, reaching 43.4 $\text{KJ}/{\text{m}}^{2}$ with the highest concentration of PLA of 1.5%. The pure sample has the lowest impact energy of 28.1 $\text{KJ}/{\text{m}}^{2}$ where it reflects the weak transmission load between the particles.

Pearson correlation analysis was performed to evaluate the relationships among tensile strength, flexural strength, and impact resistance across different composite formulations. The results are summarized in Table 4. A significant positive correlation was found between tensile strength and flexural strength (r = 0.822, *p* = 0.001), indicating that increases in tensile strength are associated with corresponding increases in flexural strength. Conversely, a strong negative correlation was observed between tensile strength and impact resistance (r = − 0.798, *p* = 0.002), suggesting that higher tensile strength corresponds to reduced impact resistance. Additionally, a moderate negative correlation was identified between flexural strength and impact resistance (r = − 0.440, *p* = 0.153); however, this relationship was not statistically significant.

**Table 4:** Pearson correlation matrix.

	Tensile strength	Flexural stress	Impact resistance
Tensile strength	1.000	0.822	− 0.798
Flexural stress	0.822	1	− 0.44
Impact resistance	− 0.798	− 0.44	1
Sig. (2-tailed)	0	0.001	0.002
N	12	12	12

Significance (2-tailed) p values < 0.05 are considered statistically significant.

In summary, the highest tensile and flexural strengths were observed in the composite containing 0.5% MWCNTs. Adding PLA reduced both tensile and flexural strength but increased elongation at break. Impact resistance improved with higher PLA content, reaching a maximum at 1.5% PLA combined with MWCNTs and Orthocryl resin. The addition of MWCNTs enhanced tensile strength by 12%, and bending strength by 59%. Impact energy is a critical property, given that heel strike is the most repetitive load during gait. The MWCNTs 0.5%/PLA 1.5% composite exhibited the highest impact energy (43.4 kJ/m^2^), indicating improved energy storage capability for the toe-off phase—an essential feature of carbon-based reinforcements such as carbon fibers or nanotubes.

### Imaging results

#### FT-IR analysis

**Fig. 7:**
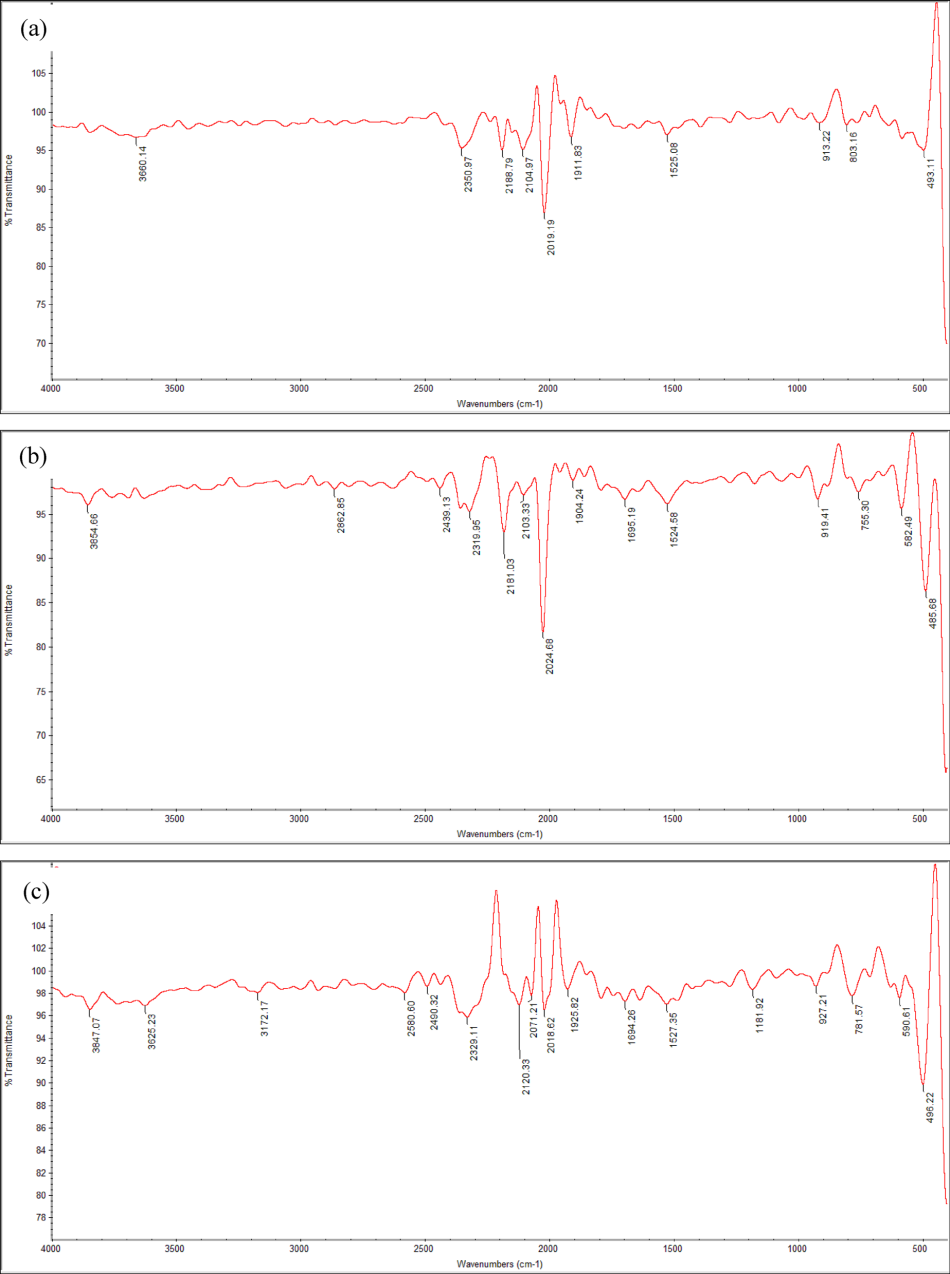

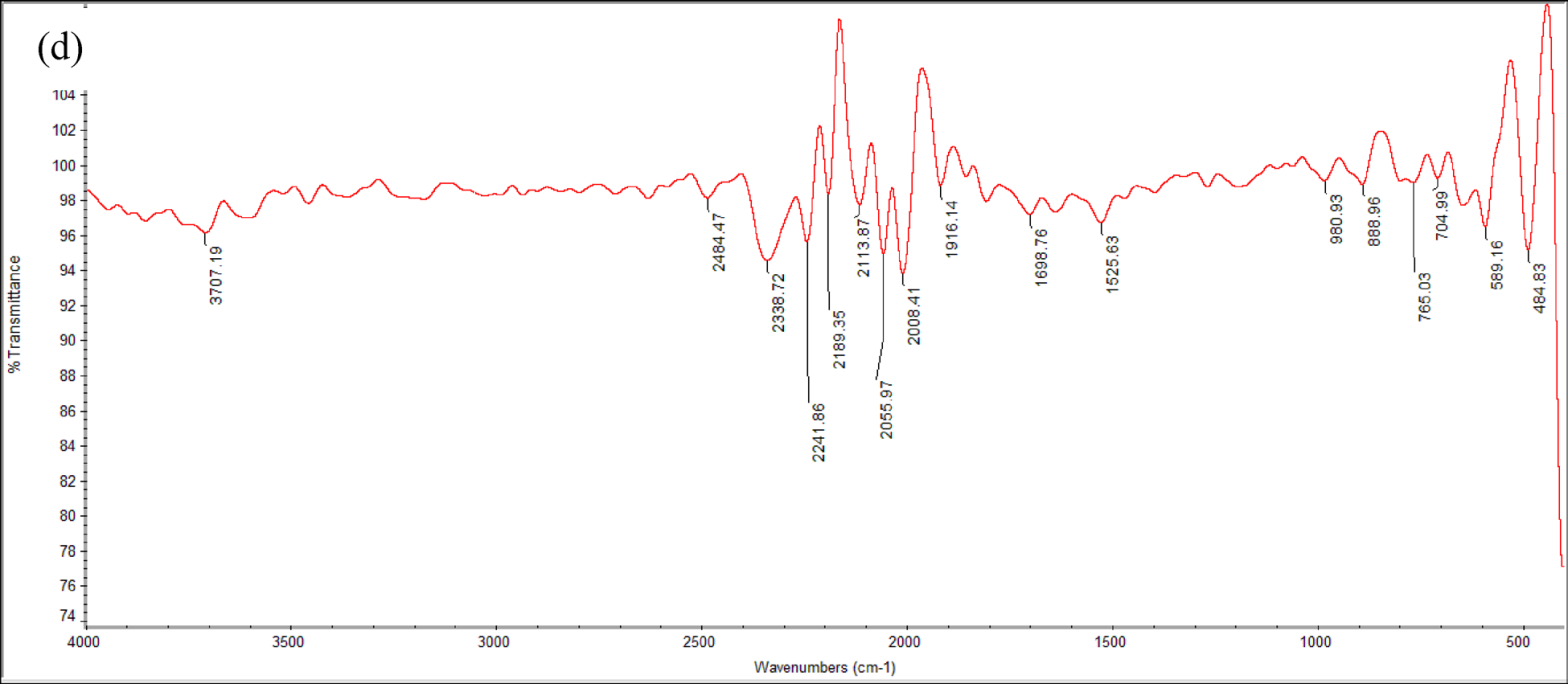
FTIR or the four composite materials, (**a**) Pure Orthocryl, (**b**) CNT0.5%, (**c**) CNT 0.5/PLA 1% and (**d**) CNT 0.5%/PLA 1.5%

FT-IR analysis was performed to analyze the functional groups and interactions present in the MWCNTs/Orthocryl composite materials at three different concentrations: CNT 0.5%, CNT 0.5%/PLA 1%, and CNT 0.5%/PLA 1.5%. Figure 7 shows several characteristic peaks. Figure 7a shows the FT-IR spectrum of pure Orthocryl, which is primarily composed of polymethyl methacrylate (PMMA). Characteristic absorption bands include ester carbonyl (C=O) stretching between 2050 and 2241 cm^−1^, methyl group C–H bending from 1380 to 1480 cm^−1^, and C–O–C bending between 1150 and 1250 cm^−1^. Additional peaks appear between 950–1050 cm^−1^ and 750–800 cm^−1^, corresponding to polymer backbone vibrations and aromatic C–H bending, respectively."

The addition of 0.5% carbon nanotubes Fig. 7b results in additional peaks at 1695 cm^−1^, 1512.4 cm^−1^, and 755 cm^−1^. These are due to the aromatic C=C stretching of CNTs and benzenoid structures, which confirms the presence of CNTs in the composite while not revealing the development of new covalent bonds. The signal at 1512.4 cm^−1^ identifies the carbon foundation of CNTs^42^. Figure 7c, d illustrate the spectra of composites with PLA added. PLA and CNTs still show their typical peaks, including C=O stretching at 2241 cm^−1^ and C–O stretching at 1305 cm^−1^. The conservation of these peaks indicates that the constituent components retain their chemical identities, and the spectrum changes are most likely caused by physical interactions and dispersion within the matrix, rather than the formation of new chemical bonds.

#### FE-SEM observations

**Fig. 8:**
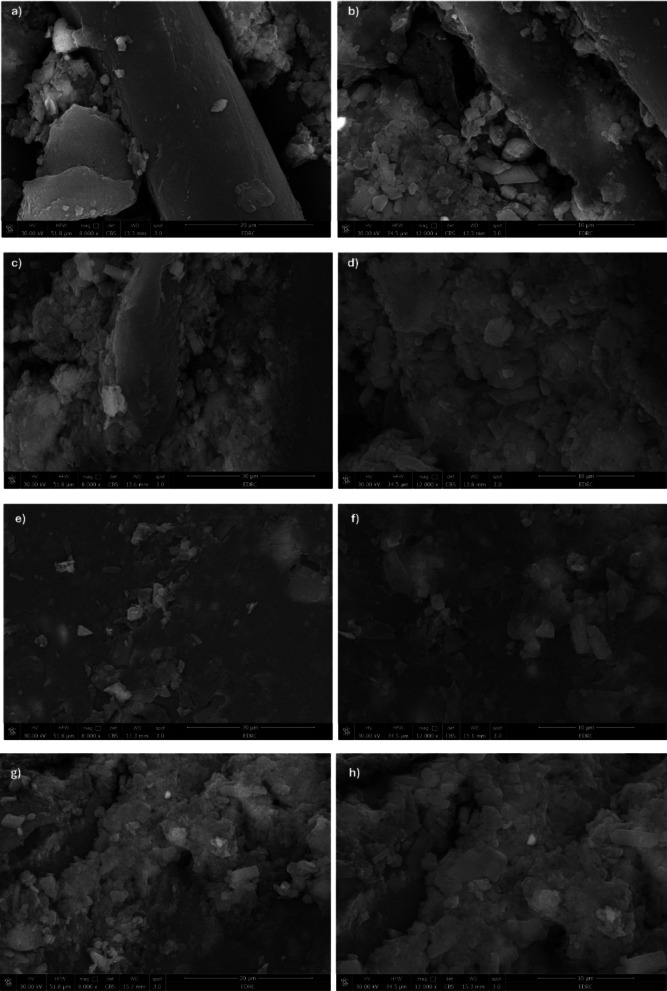
FE-SEM micrograph with ×8000 magnification of; (**a**) Pure Orthocryl, (**c**) CNTs 0.5%, (**e**) CNTs0.5%/PLA 1%, (**g**) CNTs 0.5/PLA 1.5% and FE-SEM micrograph with 12,000 × magnification of ; (**b**) Pure Orthocryl, (**d**) CNTs 0.5%, (**f**) CNTs0.5%/PLA 1%, (**h**) CNTs 0.5/PLA *1.5%.*

**Fig. 9:**
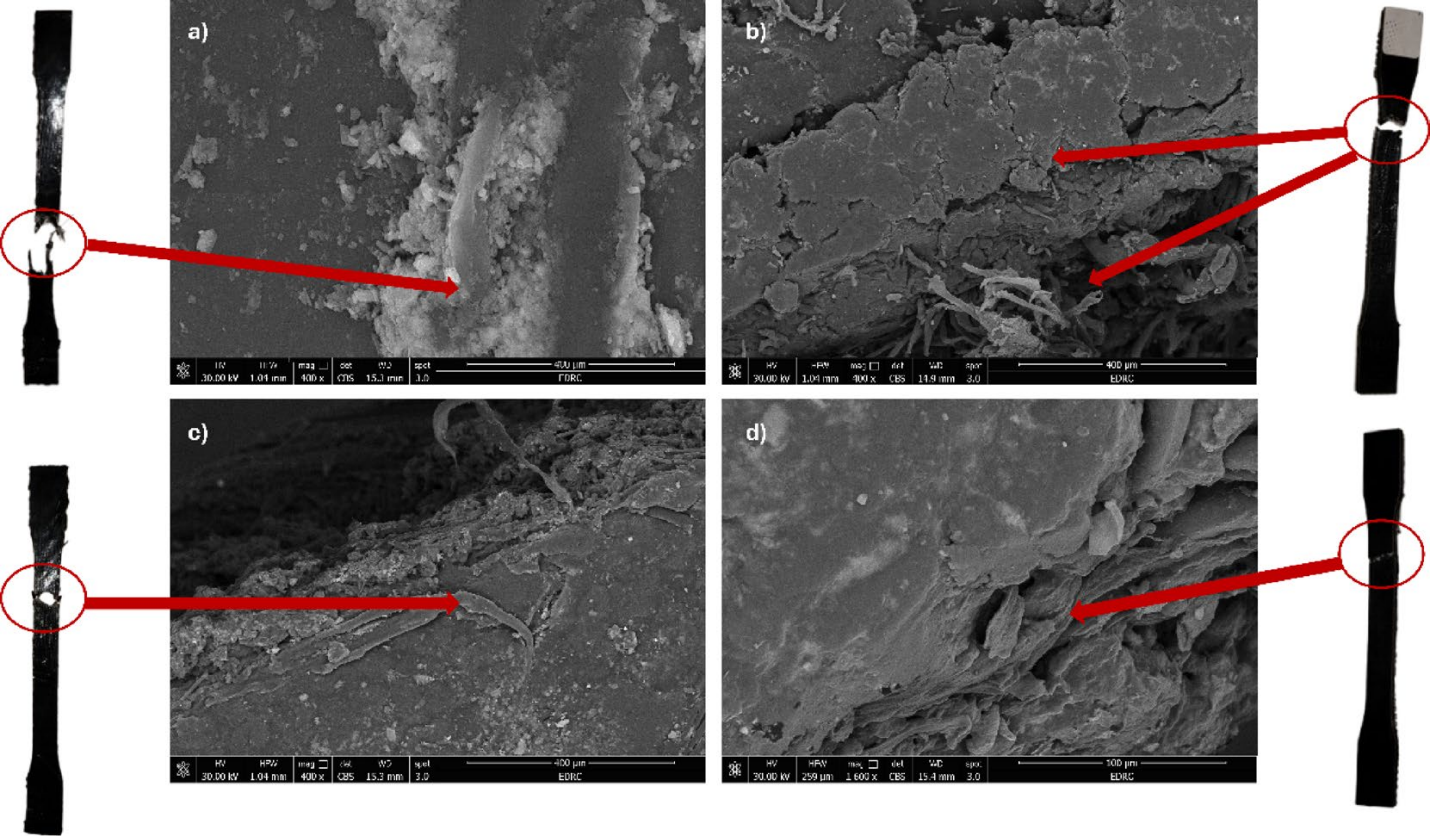
FE-SEM micrograph with magnification ×400 of cracked samples, (**a**) Pure Orthocryl, (**b**) CNTs 0.5%, (**c**) CNTs0.5%/PLA 1%, (**d**) CNTs 0.5/PLA 1.5%.

The morphology of the specimens from the impact test was studied by FE-SEM. Figure 8 illustrates the FE-SEM images with two different magnification factors of 8000× and 12,000×. Figure 8a, b, c, d, e, f, g, h shows the specimens under FE-SEM concentrations of CNTs 0.5%/PLA 1% and CNTs 0.5% /PLA 1.5% respectively. All images are coated with 1 cm gold to enhance the material’s conductivity, captured under magnification of 8000× & 12,000×. The images show the infrastructure of the filling of PLA in MWCNTs. Where the PLA is dissolved in the nanotube’s matrix. Wave transmission through the nanocomposites is blocked by the MWCNT agglomeration in Fig. 8a, b. While dispersion of PLA in samples 3 and 4 is neat and uniformly distributed due to PLA particles that accumulate on carbon fiber sheets, as illustrated in Fig. 8c, d, e, f. This demonstrates how the matrix’s MWCNT dispersion affects the characteristics of the nanocomposite. Orthocryl lamination enhances the interfacial between MWCNTs and PLA particles, where it appears in Fig. 8g, h. It was observed that MWCNTs and PLA accumulate around the carbon fibers, and this is highlighted in the crack images. A crack was made by the tensile test with a maximum applied force (3510 KN), and the cracked samples were observed in Fig. 9 with a magnification factor of 400×. Figure 9a shows a sample of pure Orthocryl under fracture, completely separated as there is no bonding between particles. Figure 9b MWCNTs of 0.5% show smoother fracture trims than the pure sample. Figure 9c of MWCNTs of 0.5% and PLA 1% indicates more adhesive particles in rupture fibers at distal points. Figure 9d of MWCNTs 0.5%/PLA 1.5% crack shows extremely smooth fiber surfaces following the fracture lines, which results from the adhesive property in PLA particles. So, the acceptable crack sample resulted in Fig. 9d with MWCNTs 0.5% and PLA1.5%.

### Finite element assessment

Effective mesh optimization is essential for achieving accurate simulation results with acceptable computational cost. A mesh convergence study was performed to assess the impact of element size on result accuracy and efficiency. Four mesh densities, with element sizes ranging from 2 to 4 mm, were evaluated^21^. Parameters such as the number of nodes and elements, maximum von Mises stress, minimum strain, and computation time were recorded for each case.

**Fig. 10:**
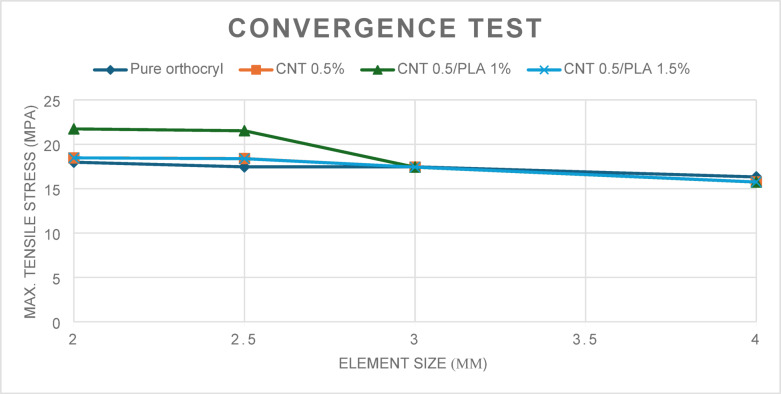
Convergence Test, Maximum Tensile Stress with element size varying from 2 to 4 mm.

As the element size decreased, the resolution of stress and strain improved, but with increased computational demands. The 2.5 mm mesh yielded a maximum von Mises stress of 17.458 MPa—comparable to the 2 mm mesh with reduced computation time and element count. It also maintained appropriate deformation behavior; with strain values close to finer meshes and higher than those from coarser ones. Thus, the 2.5 mm element size was selected as the optimal balance between accuracy and efficiency for simulating AFO material performance under load. Figure 10 shows the convergence results across all tested mesh sizes.

**Fig. 11:**
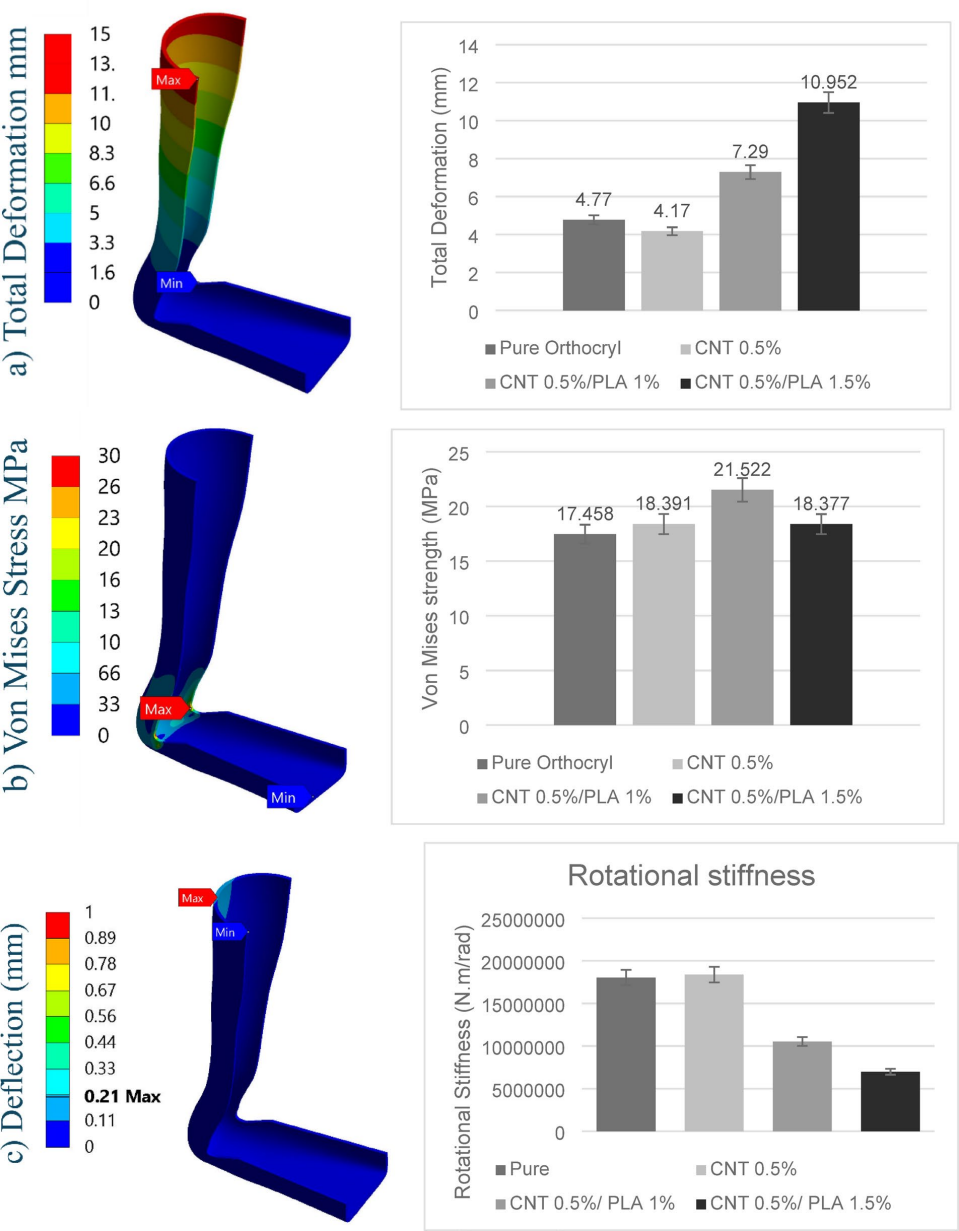
Finite Element Assessment, (**a**) Total deformation mm, (**b**) Von mises Stress MPa, (**c**) Directional Deflection in Y axis mm.

The FEA was conducted to evaluate the mechanical performance of the proposed MWCNTs/PLA/Orthocryl composites compared to pure Orthocryl. The maximum Von Mises stress and total deformation for each sample are summarized in Fig. 11 that shows the results for total deformation, von mises stress and deflection of the AFO calf.

The pure Orthocryl sample exhibited a maximum Von Mises stress of 17.458 MPa with a total deformation of 4.77 mm. Incorporating 0.5% MWCNTs improved performance, increasing the stress to 18.391 MPa while reducing the deformation to 4.17 mm, indicating enhanced load-bearing capacity and material stiffness.

For the composite containing 0.5% MWCNTs and 1% PLA, the maximum stress further increased to 21.522 MPa, representing the highest stress tolerance among the tested materials. However, total deformation also increased to 7.29 mm, indicating a reduction in stiffness compared to both pure Orthocryl and the CNT-only composite. Increasing the PLA content to 1.5% led to a slight decrease in stress to 18.377 MPa and a substantial increase in deformation to 10.95 mm, suggesting that excess PLA compromises structural rigidity despite maintaining comparable stress levels to the 0.5% CNT sample.

To further clarify the difference between stiffness and strength, rotational stiffness was calculated using the moment-to-angle ratio based on calf deflection under the applied load. The pure Orthocryl sample exhibited a rotational stiffness of 18.04 × 10⁶ $\text{N}\cdot \text{mm}/\text{rad}$, while the 0.5% CNT composite had the highest stiffness at 18.39 × 10⁶ $\text{N}\cdot \text{mm}/\text{rad}$, confirming its superior rigidity. In contrast, the 0.5% CNT/1% PLA and 0.5% CNT/1.5% PLA composites demonstrated reduced rotational stiffness, measured at 10.53 × 10⁶ $\text{N}\cdot \text{mm}/\text{rad}$ and 6.98 × 10⁶ $\text{N}\cdot \text{mm}/\text{rad}$, respectively. These findings indicate that although PLA-containing composites may exhibit higher stress levels in FEA, they experience greater angular deflection, confirming lower stiffness.

SF calculations, derived from the ratio of experimentally measured yield tensile strength to maximum Von Mises stress, further support these observations. The pure Orthocryl and 0.5% CNT samples had safety factors of 2.89 and 3.2, respectively, indicating a high margin of structural reliability. The PLA-containing composites exhibited lower safety factors of 1.13 and 1.12. Although these values remain above 1, signifying safe performance under the applied loads, they reflect a reduced safety margin.

Overall, these results emphasize that stiffness is primarily reflected by lower deformation and higher rotational stiffness, while strength is indicated by the material’s capacity to withstand stress without failure. The 0.5% CNT composite achieved a favorable balance between stiffness, strength, and safety, making it the most suitable configuration for AFO applications. In contrast, the addition of PLA increased compliance and reduced both rotational stiffness and safety factors, despite an apparent improvement in stress tolerance in some cases. This highlights the importance of evaluating both stiffness and strength when selecting materials for orthotic designs.

## Discussion

The mechanical performance of the produced composite materials was assessed using tensile, flexural, and impact resistance tests, as shown in Fig. 6. These findings demonstrate how the addition of MWCNTs and PLA altered the structural behavior of the Orthocryl matrix. The average tensile strength of pure Orthocryl was 52.79 MPa. The inclusion of 0.5% MWCNTs resulted in a significant improvement in tensile strength (59.4 MPa). This increase is due to the nanotubes’ reinforcing impact and capacity to promote effective stress transfer throughout the polymer matrix. The rise also indicates a satisfactory level of interfacial adhesion between the MWCNTs and the Orthocryl, most likely due to physical interactions and excellent dispersion, as shown by the FE-SEM study Fig. 8.

The pure Orthocryl sample has an average tensile strength of 52.79 MPa. The addition of 0.5 wt% MWCNTs boosted the tensile strength to 59.4 MPa, demonstrating the nanotubes’ effective reinforcing capabilities. This enhancement can be due to the MWCNTs’ superior dispersion and physical interactions, which enable effective stress transfer inside the polymer matrix. Flexural strength improved similarly, increasing from 52.07 MPa in the pure sample to 82.92 MPa with 0.5% CNTs.

In contrast, the addition of PLA to CNTs resulted in a decrease in both tensile and flexural strength. The average tensile strengths of the CNT 0.5%/PLA 1% and CNT 0.5%/PLA 1.5% composites decreased to 23.81 MPa and 21.95 MPa, respectively, while the flexural strengths decreased to 42.95 MPa and 38.29 MPa. The reduction in tensile and flexural strength suggests limited interfacial bonding between PLA and Orthocryl, potentially due to differences in molecular polarity and phase dispersion, leading to stress concentration points.

However, it is interesting to note that the addition of PLA considerably increased the composites’ impact resistance. The impact resistance of pure Orthocryl increased from 28.12 kJ/m^2^ to 40.9 kJ/m^2^ and 43.4 kJ/m^2^ in PLA-containing composites. This enhancement suggests increased energy absorption capacity, which is useful in applications that require more flexibility and durability.

The mechanical behavior of PLA-containing composites aligns with these criteria. While tensile and flexural strengths decreased compared to pure Orthocryl, the significant improvement in impact resistance reflects enhanced energy dissipation and shock absorption capabilities. This strength–flexibility trade-off is functionally advantageous for applications requiring comfort, compliance, and dynamic movement^38,43^.

All samples are manufactured by the casting lamination vacuum technique. Although additive manufacturing offers advanced customization, it remains cost-prohibitive and less accessible in many clinical environments, particularly in developing countries. Given that children with CP often require frequent AFO replacements due to growth, the casting method remains a practical and affordable solution.”

The correlation study revealed vital information on the dependency of mechanical properties in CNT/PLA-based composite laminates. A strong positive correlation between tensile strength and flexural strength (r = 0.822, *p* = 0.001) indicates that the structural reinforcement mechanisms that contribute to improved tensile behavior, such as effective stress transfer and matrix-filler interaction, also improve the material’s bending resistance. However, the strong negative correlation shown between tensile strength and impact resistance (r = − 0.798, *p* = 0.002) suggests a trade-off between strength and toughness. This phenomenon is typical in polymer composites, where greater stiffness and alignment of reinforcing agents improve strength while reducing the material’s ability to absorb unexpected energy due to brittleness. Overall, these findings highlight the importance of a balanced design approach in composite construction, especially when attempting to optimize both load-bearing capacity and energy absorption characteristics.

To evaluate the performance of the developed composites, Table 5 presents a comparative analysis with previous studies that employed similar lamination techniques, reinforcement strategies, and perlons layering. Specifically, when compared to earlier works using the same fabrication method and reinforcement concentrations, the present study shows a 23% increase in tensile strength and a 21.9% increase in flexural strength for the CNT 0.5% sample. Prior studies reported tensile and bending strengths of 48 MPa and 68 MPa, respectively^31,44^ while the CNT 0.5% specimen in the current study achieved 59.4 MPa and 82.92 MPa.

**Table 5:** Comparison with the standard and previous studies.

Test types	Standard range of AFOs^31,44^	Particles^31^	Sosiati^10^	Khandagale^12^	Specimens			
					Pure	CNT 0.5%	CNT 0.5%/PLA 1%	CNT0.5/PLA 1.5%
Tensile MPa	20–80 MPa	48	42	49.479	52.79	59.4	23.8	21.95
Bending MPa	45–120 MPa	68	62	Not calculated	52.07	82.92	42.95	38.29

In a related study by research in^10^, a composite of Abaca fiber (AF), epoxy (EP), and activated carbon particles (ACPs) achieved tensile and flexural strengths of 42 MPa and 62 MPa, respectively, using 20 vol% AF with varying ACP content. While those results are commendable, the current CNT-reinforced Orthocryl composite outperforms them, particularly in bending strength, highlighting the superior reinforcing potential of CNTs in polymer matrices.

Moreover, researchers in^12^ developed a laminated AFO intended for CP patients using a different ASTM standard, achieving a tensile strength of 49.48 MPa and a maximum force of 1.9 kN. Although our PLA-containing samples showed reduced strength (e.g., 23.8 MPa and 21.95 MPa), this decrease aligns with the functional requirements for CP applications, where increased flexibility and controlled stiffness are desirable to support plantar flexion and improve patient comfort during gait.

Table 5 summarizes this comparative performance, placing the present study’s results within the standard tensile and bending ranges for AFOs (20–80 MPa and 45–120 MPa, respectively). The CNT 0.5% and pure Orthocryl specimens fall well within these limits, and even the PLA-containing composites, while lower in strength, remain within acceptable ranges for specific patient-centered applications.

These findings underscore the effectiveness of MWCNTs reinforcement and the potential role of PLA in tailoring mechanical performance for orthotic applications, particularly for individuals with CP, where a balance of support and flexibility is critical.

Based on the FT-IR analysis of MWCNTs/PLA with Orthocryl composite materials at varying concentrations, several key observations can be made. The pure Orthocryl spectrum without additives, Fig. 7a displayed characteristic wavenumbers corresponding to its main component, PMMA. Notably, distinct peaks were identified, such as C–O stretching, C–H bending, and aromatic C–H bending. When 0.5% carbon nanotubes (CNTs) were introduced, Fig. 7b notable shifts in wavenumbers were observed, indicating the interaction between the CNTs and Orthocryl. Peaks at 1695, 1512.4, and 755 cm^−1^ were associated with the presence of CNTs, specifically highlighting the aromatic ring and the benzenoid stretching mode indicative of CNTs’ graphite structure.

The addition of PLA in varying concentrations Fig. 7c, d showed that the materials maintained their distinct wavenumbers, demonstrating that each component preserved its structural integrity within the composite. Common peaks between PLA and Orthocryl, particularly at 2241 cm^−1^ for C=O stretching and 1305 cm^−1^ for C–O stretching, suggest a synergistic combination of these materials. Overall, the FT-IR spectra indicate the successful integration of MWCNTs and PLA with Orthocryl, preserving the unique characteristics of each material while forming a cohesive composite structure. The observed shifts and overlaps in wavenumbers highlight the interactions and compatibility of these components, paving the way for potential enhancements in the composite’s properties.

FE-SEM analysis was performed to examine the morphology of the composites. The results revealed a uniform dispersion of PLA within the MWCNT matrix. Orthocryl lamination improved the interfacial adhesion between MWCNTs and PLA, contributing to enhanced mechanical performance. Figure 8 presents FE-SEM images at ×8000 and ×12,000 magnifications for composites containing CNTs 0.5%/PLA 1% and CNTs 0.5%/PLA 1.5%. The images show the structural integration of PLA within the MWCNT matrix. They reveal the structural integration of PLA within the MWCNTs matrix. Here, PLA dissolves into the nanotube matrix by MWCNTs agglomeration in Fig. 8e, f. Conversely, Fig. 8g, h show a more uniform PLA dispersion due to PLA particles accumulating on carbon fiber sheets, highlighting the significant impact of MWCNTs dispersion on the nanocomposite’s properties. Orthocryl lamination is noted for enhancing the interface between MWCNTs and PLA particles.

The fracture surfaces of the composites, shown in Fig. 9, reveal how the addition of MWCNTs and PLA affected the material’s fracture resistance and bonding. In pure Orthocryl Fig. 9a, the lack of interfacial bonding caused a brittle failure with complete separation. However, 0.5% MWCNTs Fig. 9b led to smoother fracture edges, indicating improved stress transfer and reduced brittleness. When PLA was added Fig. 9c, 1% PLA, better adhesion was observed at the fracture points, enhancing ductility and crack resistance. The best fractured performance occurred with 0.5% MWCNTs and 1.5% PLA Fig. 9d which exhibited smooth fracture surfaces and strong interfacial bonding, resulting in controlled failure and improved flexibility. These findings suggest that MWCNTs reinforce the composite, while PLA enhances adhesion and flexibility, making this combination ideal for orthotic applications.

The FEA was used to assess the mechanical behavior of several composite materials under loading circumstances like those found in AFOs. The selected 2.5 mm mesh provided a practical balance between computational efficiency and accuracy, supporting reliable simulation of AFO material behavior. This choice ensured sufficient resolution in stress and strain distribution, particularly in critical regions, without incurring excessive processing time, an essential consideration in iterative design and optimization processes.

The metrics studied were Von Mises stress is considered a measure of yield strength, while the total deformation is a validation of flexibility and energy absorption^45^. The FEA results show that modified Orthocryl composites have promising mechanical performance for use in AFOs particularly for children with CP who require customized support to improve gait stability, reduce spasticity effects, and increase mobility^46,47^ While all material samples maintained similar Von Mises stress values (~ 17.42 MPa), indicating sufficient structural strength for load bearing in orthotic use, the total deformation varied significantly, highlighting differences in flexibility and shock absorption—both crucial factors for children.

SF is a critical parameter in AFO design to ensure durability and safe performance under patient-specific loads, particularly in pediatric applications. All tested composites-maintained SF values above 1, confirming structural adequacy. The CNT 0.5% composite achieved both high strength and a favorable safety margin. The PLA-reinforced composites demonstrated increased flexibility while maintaining acceptable safety factors, making them suitable for applications that prioritize user comfort and material adaptability^48^.

In terms of deformation, the pure Orthocryl and CNT 0.5% composites exhibited lower displacement values (4.77 mm and 4.17 mm), indicating higher stiffness, which may limit dynamic movement. Conversely, the CNT 0.5%/PLA 1% and CNT 0.5%/PLA 1.5% composites showed greater deformation (7.28 mm and 10.93 mm), reflecting enhanced flexibility and improved energy absorption, which can be beneficial for comfort during prolonged use.

Rotational stiffness was also evaluated to quantify the AFO’s resistance to angular deformation, which directly affects postural stability during use. The pure Orthocryl sample exhibited a rotational stiffness of 18.04 × 10⁶ $\text{N} \text{mm}/\text{rad}$, while the 0.5% CNT composite showed a slightly higher value of 18.39 × 10⁶ $\text{N} \text{mm}/\text{rad}$, indicating increased structural rigidity. In contrast, the composites containing PLA demonstrated substantially lower rotational stiffness values: 10.53 × 10⁶ $\text{N} \text{mm}/\text{rad}$ for the 0.5% CNT/1% PLA composite and 6.98 × 10⁶ $\text{N} \text{mm}/\text{rad}$ for the 0.5% CNT/1.5% PLA composite.

These findings confirm that the addition of PLA increases compliance and reduces rotational stiffness, even when FEA results show higher stress tolerance. Rotational stiffness serves as a critical indicator of the AFO’s ability to maintain stability and resist bending during use, complementing the strength and SF analysis^49^. Among the tested materials, the CNT 0.5%/PLA 1% composite demonstrated the most favorable balance between strength and flexibility, combining higher stress capacity with controlled deformation and an acceptable safety margin. This balance is essential for pediatric AFO applications, where providing sufficient support while enhancing comfort can improve long-term compliance and reduce fatigue associated with rigid orthotic designs.

These findings confirm that the addition of PLA increases compliance and reduces rotational stiffness, even when FEA results show higher stress tolerance. Rotational stiffness serves as a critical indicator of the AFO’s ability to maintain stability and resist bending during use, complementing the strength and SF analysis^49^. Among the tested materials, the CNT 0.5%/PLA 1% composite demonstrated the most favorable balance between strength and flexibility, combining higher stress capacity with controlled deformation and an acceptable safety margin. This balance is essential for pediatric AFO applications, where providing sufficient support while enhancing comfort can improve long-term compliance and reduce fatigue associated with rigid orthotic designs.

Table 6 illustrates comparison to previous studies^37^, the proposed materials show lower Von Mises stress and enhanced flexibility, crucial for reducing spasticity and improving mobility. The CNT 0.5%/PLA 1.5% composite stands out for its ability to support dynamic movement while ensuring comfort and long-term use in pediatric AFOs.

**Table 6:** FEA for the proposed materials.

	Pure Orthocryl	CNT 0.5%	CNT 0.5%/PLA 1%	CNT 0.5%/PLA 1.5%	^37^
Von misses MPa	17.458	18.391	21.522	18.378	40.41
Total deformation mm	4.77	4.17	7.29	10.95	13.03
Safety factor	2.89	3.2	1.33	1.13	Not calculated

### Study limitations and future work

This study was limited to the fabrication and mechanical characterization of composite material samples for potential use in AFOs. Full-scale AFO devices were not produced, and clinical evaluations such as gait analysis or patient trials were not conducted. Although PLA was incorporated as a biodegradable component to enhance sustainability, no life cycle assessment was performed; thus, sustainability was inferred from material selection rather than quantitatively evaluated.

A limitation of this study is the use of manually measured strain data to calculate Poisson’s ratio for input into the finite element analysis. Future work will incorporate extensometers or digital image correlation for more accurate strain characterization. Furthermore, the finite element analysis was limited to static loading conditions, which may not fully represent the dynamic mechanical behavior of AFOs during actual use. Future work should focus on developing functional AFO prototypes, conducting dynamic simulations, and performing clinical gait analysis assessments for pediatric children to validate performance and usability in real-world settings.

## Conclusion

This study investigated the development of enhanced materials used in AFO by integrating MWCNTs and PLA into Orthocryl using a laminated manufacturing technique. The process involved vacuum-assisted layering of perlons and carbon fiber sheets, followed by precision cutting with CNC laser technology.

Mechanical characterization demonstrated notable improvements in the composites’ performance. The addition of 0.5% MWCNTs increased tensile strength by 12% and bending strength by 59%, confirming the reinforcing effect of CNTs. In contrast, adding 1% and 1.5% PLA reduced tensile strength but significantly increased elongation at break and impact resistance, indicating enhanced ductility. The 0.5% MWCNTs/1.5% PLA composite achieved the highest impact energy of 43 kJ/m^2^, suggesting suitability for applications requiring higher flexibility and shock absorption.

Chemical analysis using FT-IR confirmed effective integration of MWCNTs and PLA into the Orthocryl matrix, with characteristic wavenumber shifts indicating good interfacial compatibility. FE-SEM analysis further validated the uniform dispersion of CNTs and PLA, revealing improved interfacial adhesion and enhanced fracture behavior, contributing to the composite’s mechanical performance.

The FEA was employed to simulate the mechanical behavior of the composites in AFO applications. A 2.5 mm mesh size was identified as the optimal balance between computational efficiency and result accuracy. The simulations demonstrated that the 0.5% CNT/1% PLA composite provides a favorable combination of strength, flexibility, and safety factor, while the 0.5% CNT composite offers higher stiffness and load-bearing capacity. These findings indicate that modifying Orthocryl with CNTs and PLA enables the tailoring of mechanical properties to meet different functional requirements.

Overall, this work provides a foundation for the development of customized AFO materials with balanced strength, flexibility, and safety. Future research will extend this analysis to clinical applications and dynamic gait conditions.

## Supplementary Information

Below is the link to the electronic supplementary material.

Supplementary Material 1

## Data Availability

All data generated or analyzed during this study are included in this published article [and its supplementary information files].
